# *Trypanosoma cruzi* infection in pregnancy: New approaches to address the diagnosis and follow-up of women and newborns

**DOI:** 10.1371/journal.pntd.0014665

**Published:** 2026-08-28

**Authors:** Yves Carlier, Jackeline Alger, Eric Dumonteil, Claudia Herrera, Alejandro G. Schijman, Pierre Buekens

**Affiliations:** 1 Department of Tropical Medicine and Infectious Disease, Celia Scott Weatherhead School of Public Health and Tropical Medicine, Tulane University, New Orleans, Louisiana, United States of America; 2 Laboratory of Parasitology, Faculty of Medicine, Université Libre de Bruxelles (ULB), Brussels, Belgium; 3 Executive Direction, Instituto de Enfermedades Infecciosas y Parasitología Antonio Vidal, Tegucigalpa, Honduras; 4 Laboratorio de Biología Molecular de la Enfermedad de Chagas, Instituto de Investigaciones en Ingeniería Genética y Biología Molecular “Dr Héctor Torres” (INGEBI-CONICET), Buenos Aires, Argentina; 5 Department of Epidemiology, Celia Scott Weatherhead School of Public Health and Tropical Medicine, Tulane University, New Orleans, Louisiana, United States of America; Oswaldo Cruz Foundation: Fundacao Oswaldo Cruz, BRAZIL

## Abstract

The transplacental route is the main human-to-human transmission mode of *Trypanosoma cruzi* infection, causing congenital Chagas disease, a major health problem in most endemic and nonendemic areas. The prevention, diagnosis, and follow-up of pregnant women and congenitally infected newborns face limitations, such as regional variations of concordance among serological tests used to diagnose *T. cruzi* infection in the Americas, the low sensitivity of microscopic examination for detecting live trypomastigotes in blood, and losses of infected women and newborns in screening and follow-up programs. In addition, fetal growth retardation was recently documented in uninfected newborns of infected mothers. The potential practical impacts of some new approaches, based on fundamental concepts and results of clinical observations addressing *T. cruzi* infection in pregnancy, such as simplifying the serodiagnosis, detecting and identifying live *T. cruzi* clones and monitoring fetal growth retardation in all neonates of infected mothers, are discussed as open research questions.

## Introduction

The protozoa parasite *Trypanosoma cruzi* is the etiological agent of Chagas disease (CD). The transplacental (congenital) route is the main human-to-human transmission mode of *T. cruzi* infection in most endemic and nonendemic areas. Roughly 5% of infected pregnant women in the chronic phase of *T. cruzi* infection give birth to congenitally infected newborns [1]. Besides a few fatal cases, some infected neonates develop clinical signs, whereas others display asymptomatic infections [2]. When untreated, congenital Chagas disease (CCD) can progress later in life towards chronic severe and fatal CD (such as Chagas cardiomyopathy) among 20–40% of subjects. Diagnosis of chronic infection in pregnant women is mainly based on detection of *T. cruzi*-specific antibodies. Diagnosis of acute infection in newborns is based either on detection of *T. cruzi* in blood (by microscopic examination) and/or detection of parasitic DNA (mainly using PCR) and/or serological testing in infants older than 8–10 months of age (i.e., after elimination of passively transferred maternal antibodies) [3]. Treatment of congenital *T. cruzi* infection is well tolerated and nearly 100% effective using one of both trypanocidal options, benznidazole (BN) or nifurtimox (NF), if administered in the year after birth. Moreover, prevention of congenital transmission might be achieved by treating infected girls and nonpregnant women with BN or NF [4], whereas such treatment cannot be applied during pregnancy and breastfeeding. These considerations form the basis for the current public health recommendations for prevention, diagnosis, treatment, and follow-up of congenitally infected newborns and pregnant women [5–7].

**Fig 1:**
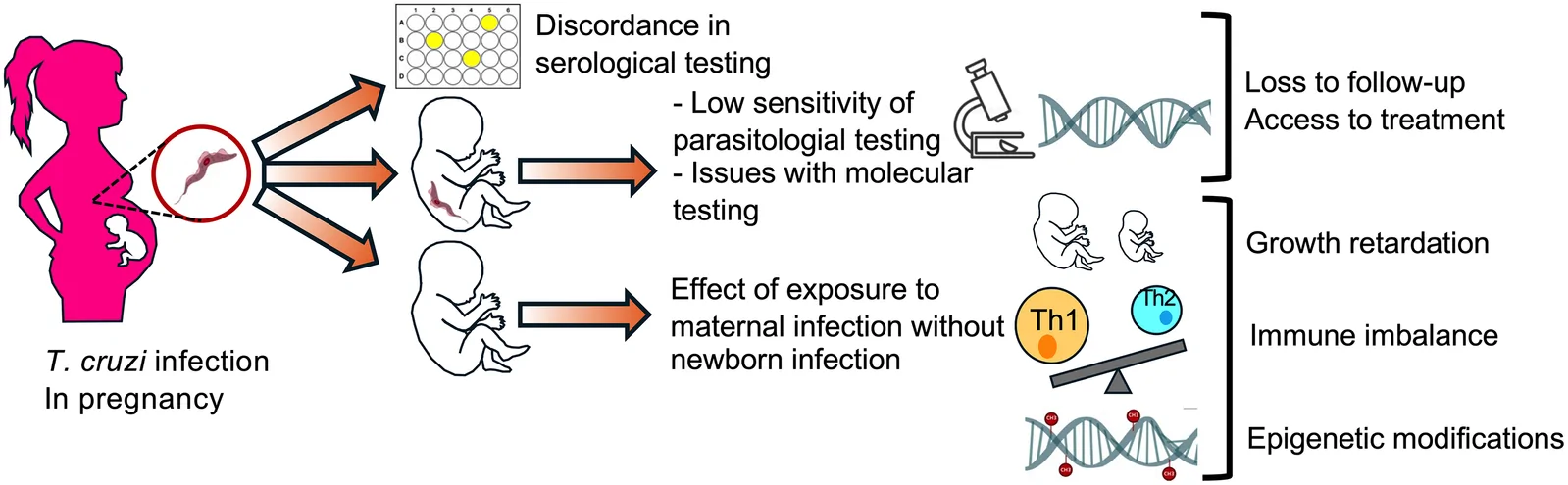
*Trypanosoma cruzi* infection in pregnancy.

However, the poor concordance between serological tests to diagnose *T. cruzi* infection observed in some regions of the Americas and the low sensitivity of microscopic examination to detect moving trypomastigotes in blood result in losses in screening and follow-up of infected women and newborns [3,8,9]. This viewpoint aims to discuss the potential and practical impacts of a recently highlighted fundamental concept (mainly the harmful association(s) of *T, cruzi* clones that would favor the congenital transmission of parasites) and new clinical observations relevant to the diagnosis and follow-up of newborns and women when *T. cruzi* infection occurs during pregnancy (Fig 1).

## Simplifying the serodiagnosis of *T. cruzi* infection for screening and prevention of CCD

Serological screening of all pregnant women aims to identify infected infants who could be effectively cured early in life. However, discrepancies are frequently observed between the results of currently available serological tests (mainly in Central and North America; [8]), often requiring a third confirmatory test to establish the definitive diagnosis, in agreement with the WHO/PAHO/CDC recommendations [5–7]. Such recommendations mainly intend to reduce false positives and avoid unnecessary treatment with trypanocidal drugs causing adverse effects in adults. However, discrepancies might result from the lack of some antigens specific to *T. cruzi* clones infecting the host in the serological assays, highlighting the possibility of false negative results in detecting antibodies (rather than false positive) [10].

So, as previously highlighted [11], a simplification of the procedure of *T. cruzi* serodiagnosis for pregnant women, considering only one positive result if two tests are used, is proposed. Going a little further, only one test might be used in regions of the Americas where the serological concordance is high, relying on the test validated as having a higher reported sensitivity in the region. These approaches would avoid wasting of scarce resources, streamline the screening cascade by reducing the need for confirmatory testing, shortening sample-processing times, and minimizing the risk of losing infected pregnant women during follow-up. It would also accelerate access to treatment for infected children. In addition, this new algorithm would provide important benefits for the serodiagnosis of 8–10-month-old infants born to infected mothers, as well as for infected girls and nonpregnant women, with the aim of preventing future congenital transmission. There is an urgent need for real-world evidence supporting this simplified approach.

## Detection of live *T. cruzi* parasites by RT-PCR to increase accuracy of the diagnosis of CCD

The detection of moving trypomastigotes in blood (by microscopic examination, mainly using the microhematocrit test) confirms the diagnosis of congenital *T. cruzi* infection. However, this method displays relatively low sensitivity and nowadays PCR, displaying higher sensitivity than microscopic examination [3,9], is more frequently used for such diagnosis mainly in nonendemic settings, pending LAMP validation to facilitate molecular diagnosis also in resource-limited endemic regions [3]. However, these assays only detect circulating parasitic DNA, which remains stable for days after parasite lysis [12],

Indeed, most *T. cruzi*-infected pregnant women arm their fetus by inducing a significant proinflammatory immune response in blood cells of uninfected newborns and transmitting IgG antibodies, able to opsonize trypomastigotes, promoting their phagocytosis and killing by cells expressing FcγR [13,14]. So, it cannot be excluded that some live parasite clones transmitted trans-placentally might be prevented from further development toward CCD through the maternally induced armed fetal/neonatal immune response. Such self-cured congenital infection might lead to the release of DNA from killed parasites and PCR/LAMP positive results in cord or neonate blood, although not corresponding to an actual congenital *T. cruzi* infection.

Interestingly, RNA is a molecular marker of pathogen viability, since it is degraded when parasite lysis occurs [12]. Reverse-transcription quantitative real-time PCR (RT-qPCR) could be used to assess mRNA expression as a marker of viable *T. cruzi* parasites, as previously applied to the detection of live parasites in contaminated beverages and insect vectors [12,15]**.** This would be a valuable decision support in starting the currently long treatment regimen (to be administrated for 1–3 months) shortly after birth only in neonates displaying without any doubt a congenital infection. Standard PCR cannot provide such certainty, which can only be achieved through a serological testing to be performed only 8–10 months later, thus delaying treatment. This more precise diagnostic tool would stimulate mother adhesion and compliance with the treatment and limit the risks of refusal or premature discontinuation of treatment to infected newborns. A future study comparing the results of standard qPCR/LAMP and qRT-PCR/RT-LAMP in neonates of infected mothers would help assess the frequency and biological relevance of this potential self-cure phenomenon.

## The search for fetal growth retardation in uninfected newborns of infected mothers to complement the surveillance program of *T. cruzi* infection in pregnancy

A recent large cross-sectional study shows that uninfected newborns of *T. cruzi*-positive women have lower birth weights and smaller head circumferences than newborns from uninfected pregnant women [16]. The absence of infection in such newborns was confirmed by negative microscopic examinations (microhematocrit test) in blood from umbilical cord and 4–8 weeks old newborns, negative PCR in cord blood, and negative double serological testing at 10 months. The data include mothers and neonates from North to South America (Mexico, Honduras, and Argentina), strengthening the generalizability of this effect [16]. If fetal growth retardation is a classical observation in CCD [2], limited data are available on birth outcomes from *T. cruzi*-infected women in the absence of congenital transmission.

The mechanisms involved in such effect might be related to maternal-fetal epigenetic reprogramming observed in the studied cohort [17], in agreement with another study in *T. cruzi*-positive women who did not transmit parasites to their offspring, showing a downregulation of the KISS1 gene encoding kisspeptin, whose weak expression is associated with intrauterine growth restriction [18].

It is unclear at this stage whether such uninfected newborns with fetal growth retardation [16] have long-term consequences of being born from *T. cruzi*-infected mothers, given they are considered healthy and typically not monitored. So, a longitudinal study of uninfected newborns of *T. cruzi*-infected mothers, in addition to the congenitally infected ones, would be of interest since maternal-fetal epigenetic reprogramming/imprinting is known to permanently influence the risks for adverse health outcomes in infancy and adulthood [19].

## Identification of cruziomes infecting pregnant women to help identify neonates at higher risk of congenital infection or other adverse outcomes

As recently proposed, “cruziome” defined the set of *T. cruzi* clones and their variants infecting a given host [10]. Various cruziomes are commonly observed in pregnant women and some of them might be preferentially associated with congenital infection, highlighting the need to re-evaluate the relationships between parasite diversity during pregnancy and its consequences for the newborns [10]. Indeed, some harmful maternal cruziomes, might be more likely to cross the placenta, as occurs in the 5% of infected pregnant women who transmit infection to their fetus. By contrast, other cruziomes might not be transmitted, as occurs in 95% of infected pregnant women, but nevertheless induce maternal epigenetic/imprinting effects in uninfected newborns (less harmful cruziomes) or be without any effect (nonharmful cruziomes) [10].

Identifying harmful and nonharmful cruziomes using genomic, transcriptomic, proteomic and/or immunomic methods remains currently a research challenge for population-based studies of CD. However, the confirmation of an association between some specific maternal cruziomes and congenital family transmission and/or other negative outcomes in infancy (such as the fetal growth retardation), would allow to pay more attention to smaller groups at risk of pregnant women and newborns (those displaying harmful cruziomes), potentially improving the effectiveness of diagnosis and follow-up programs.

## Conclusion

The approaches mentioned above would: i) improve adherence to screening and compliance with the prevention and control programs of infection during pregnancy, by simplifying the algorithm of serological diagnostic for *T. cruzi* infection; ii) introduce the surveillance of neonatal epigenetic/imprinting effects of maternal infection on uninfected newborns (particularly with respect to growth); iii) focus more attention to the pregnant women and neonate groups at risk, by introducing potential new tools of precision medicine, such as the identification of harmful cruziomes and the research of live parasites. All these new approaches would be beneficial to programs addressing *T. cruzi* infection during pregnancy, particularly for the 1.7 million infected women of reproductive age and the 9,000 cases of CCD estimated in the 21 endemic countries of the Americas [20], and the 40,000–43,000 infected women and 22–315 infected infants in the United States [7,21]. They would accelerate progress towards the WHO’s 2030 goal of eliminating transmission and preventing CCD [22], provided that large-scale multinational funded studies are conducted to answer these research questions.
